# Joint screening of ultrahigh dimensional variables for family-based genetic studies

**DOI:** 10.1186/s12919-018-0120-2

**Published:** 2018-09-17

**Authors:** Subha Datta, Yixin Fang, Ji Meng Loh

**Affiliations:** Department of Mathematical Sciences, New Jersey Institute of Technology, 323 Dr. Martin Luther King Jr. Blvd, Newark, NJ 07102 USA

## Abstract

**Background:**

Mixed models are a useful tool for evaluating the association between an outcome variable and genetic variables from a family-based genetic study, taking into account the kinship coefficients. When there are ultrahigh dimensional genetic variables (ie, *p* ≫ *n*), it is challenging to fit any mixed effect model.

**Methods:**

We propose a two-stage strategy, screening genetic variables in the first stage and then fitting the mixed effect model in the second stage to those variables that survive the screening. For the screening stage, we can use the sure independence screening (SIS) procedure, which fits the mixed effect model to one genetic variable at a time. Because the SIS procedure may fail to identify those marginally unimportant but jointly important genetic variables, we propose a joint screening (JS) procedure that screens all the genetic variables simultaneously. We evaluate the performance of the proposed JS procedure via a simulation study and an application to the GAW20 data.

**Results:**

We perform the proposed JS procedure on the GAW20 representative simulated data set (*n* = 680 participant(s) and *p* = 463,995 CpG cytosine-phosphate-guanine [CpG] sites) and select the top *d* = ⌊*n*/ log(*n*)⌋ variables. Then we fit the mixed model using these top variables. Under significance level, 5%, 43 CpG sites are found to be significant. Some diagnostic analyses based on the residuals show the fitted mixed model is appropriate.

**Conclusions:**

Although the GAW20 data set is ultrahigh dimensional and family-based having within group variances, we were successful in performing subset selection using a two-step strategy that is computationally simple and easy to understand.

## Background

Compared with genome-wide DNA sequence variance investigation of blood lipids, genome-wide epigenetic investigation has been far less explored. To fill this gap, the Genetics of Lipid Lowering Drugs and Diet Network (GOLDN) study conducted an epigenome-wide association study to uncover epigenetic factors influencing lipid metabolism [1].

GAW20 provides a unique opportunity for us to analyze the real data from the GOLDN study, as well as the simulated data based upon it. Along with the opportunity come the challenges. First, the number of genetic variables is ultrahigh. The GAW20 data consists of cytosine-phosphate-guanine dinucleotide (CpG) variables, whose sizes are much larger than the number of subjects. Second, the subjects are not independent; instead, the subjects are correlated within families. Third, there are repeated measurements of the methylation and triglyceride (TG) levels. The pregenomethate values are measured at visits 1 and 2, and the postgenomethate values are measured at visits 3 and 4.

Irvin et al. [2] used mixed models to analyze the GOLDN data, using a random effect for family structure. Specifically, at each CpG site, they fitted a mixed effect model to examine its effect on the log of fasting TG level, adjusting for some fixed effects such as age and gender. Based on these marginal effects, four CpG sites in intron 1 of CPT1A were very strongly associated with TG. Actually, this marginal screening procedure is called *sure independence screening* (SIS) [3]. However, the SIS procedure may fail to identify marginally unimportant but jointly important genetic variables. Therefore, in this article, we propose a joint screening (JS) procedure that performs screening on all the genetic variables simultaneously.

We apply the proposed JS procedure to the representative simulated data set provided by GAW20. This data set is made up of the 200 simulated data sets generated by GAW20 based on the GOLDN study data [2], simulating what might happen if we were to repeat the GOLDN clinical trial, but using a new fictitious drug, called “genomethate,” that has a pharmacoepigenetic effect on the TG level.

In the representative data set, there were 717 participants in pedigrees; participants already on any lipid-lowering medication were taken off drug for a “washout period.” At visit 1 (after the washout), participants were measured after an overnight fast with a standard lipid profile. The next day, they returned to the clinic, again fasting, for a second, repeat lipid profile. All participants were then given the genomethate drug for a 3-week treatment period, after which they returned to the clinic for 2 consecutive days of lipid profiling (visits 3 and 4, both with overnight fasting), to assess the response to treatment. We considered the difference in the TG level (the original scale or the log scale) between visit 4 and visit 2 as the *outcome variable*. There were 680 participants with the observed outcome.

## Methods

### Mixed models for family data

**Fig. 1:**
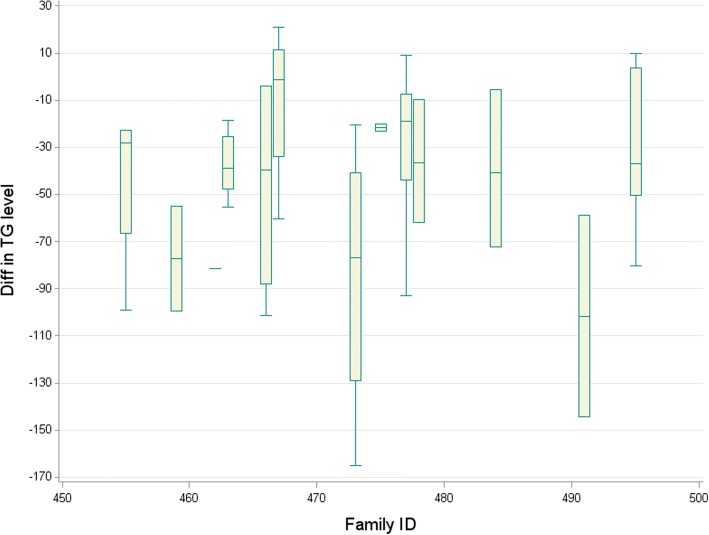
Boxplots of TG level by Pedigree number. The boxplots demonstrate that the response values vary between and within pedigrees

Mixed model analysis provides a general, flexible approach when dealing with correlated data [4]. Mixed models allow a wide variety of variance-covariance structures to be explicitly modeled. Therefore, mixed models are a useful tool to analyze the GAW20 data, because participants within the same family are correlated with each other via genetic structure. Figure 1 shows side-by-side boxplots of the outcome variable (the difference in TG level between visit 4 and visit 2) within 13 pedigrees, demonstrating the heterogeneity of the outcome variable.

Suppose that there are *n* subjects participants from a family study and there are *p* genetic variables. Assume that we can relate the phenotypes with the genetic variables via the following mixed model,

1 $$ \mathbf{Y}= X\beta +\alpha +\varepsilon $$

where **Y** is an *n* × 1 vector of observed phenotypes, *X* is an *n* × *p* design matrix of genetic variables, *β* is a *p* × 1 vector representing the fixed effects of genetic variables, and *α* = (*α*_1_, ⋯, *α*_*n*_)^′^ is an *n* × 1 vector representing the random effects. We assume that *ε* has zero-mean and $Var\left(\ \varepsilon\ \right)={\sigma}_e^2{I}_n$, and

$$ \alpha \sim N\left(0,{\sigma}_g^2K\right) $$

where *n* × *n* matrix *K* = (*k*_*ij*_)_*n* × *n*_ is the kinship matrix among the *n* participants from the family data. The kinship coefficent *k*_*ij*_ is a measure of genetic relatedness between two individuals *i* and *j*.

If *p* were small compared with *n*, we would estimate the unknown parameters,$\beta, {\sigma}_e^2$ and ${\sigma}_g^2$, in the above mixed model and then identify those genetic variables that are significantly associated with the phenotype; that is, to identify those CpG sites that are associated with the TG level.

Specifically, if *p* were small compared with *n*, we could estimate the coefficient vector *β* and the covariance matrix *Y* ,

2 $$ V= Var\left(\ Y\ \right)={\sigma}_g^2K+{\sigma}_e^2{I}_n $$

via the weighted least-squares,

3 $$ {\widehat{\beta}}_{WLS}={\left({X}^{\prime }{\widehat{V}}^{-1}X\right)}^{-1}{X}^{\prime }{\widehat{V}}^{-1}\mathbf{Y} $$

and the restricted maximum likelihood (REML),

4 $$ \widehat{V}=\mathrm{argmax}\left\{{l}_p(V)-\log |{X}^{\prime }{V}^{-1}\mathbf{X}|\right\} $$

where $$ {l}_p(V)=-\left\{\log |V|+{\left(\mathbf{Y}-X\widehat{\beta}\right)}^{\prime }{V}^{-1}\left(\mathbf{Y}-X\widehat{\beta}\right)\right\}. $$

### Curse of dimensionality

However, when the dimension of the genetic variables is ultrahigh (*p* ≫ *n*), as in the GAW20 data, we cannot use the above estimates (3) and (4) for *β* and *V*, respectively. This is an example of curse of dimensionality; the matrix under inverse in equation (3), ${X}^{\prime }{\widehat{V}}^{-1}X$, is a *p* × *p* matrix, but its rank is at most *n*. There are two reasons the classical mixed model is not working. First, the matrix ${X}^{\prime }{\widehat{V}}^{-1}X$ is not invertible, so the solution to equation (3) is not unique. Second, when *p* is ultrahigh, the computation of general inverse of ${X}^{\prime }{\widehat{V}}^{-1}X$ is very hard, not to mention the estimation of *V* in equation (4).

If the dimensional of genetic variable is high (*p*~*n* or *p* > *n*), we can use some regularization methods. These methods simultaneously estimate parameters and perform variable selection by penalizing a loss function with the help of a sparsity inducing penalty. For examples, see Tibshirani (LASSO [least absolute shrinkage and selection operator]) [5]; Hoerl and Kennard (Ridge regression) [6];Fan and Li (SCAD) [smoothly clipped absolute deviation] [7]; Zou and Hastie (elastic net) [8]; and Schelldorfer et al. [9]. However, in ultrahigh dimensional cases, the computation cost for these regularization methods becomes a concern.Therefore, for the situation with ultrahigh dimensional genetic variables, we propose a two-stage approach.

In the first stage, we conduct screening to identify a subset of genetic variables that are suspected to be associated with the outcome; choosing the subset size such that it is manageable by mixed models. In the second stage, we conduct mixed model analysis using those genetic variables that survive the screening stage. In the following two subsections, we describe these two stages in detail.

### Stage 1: A novel JS procedure

Our JS procedure for mixed models is motivated by the JS procedure for linear models proposed by Wang and Leng [10]. The JS procedure proposed by Wang and Leng [10] is called high-dimensional ordinary least-squares projection (HOLP) and is for the following linear model,

5 $$ \overset{\sim }{Y}=\overset{\sim }{X}\beta +\overset{\sim }{\varepsilon } $$

where $\overset{\sim }{Y}$ is an *n* × 1 vector of observed phenotypes, $\overset{\sim }{X}$ is an *n* × *p* design matrix of genetic variables, and *β* is a *p* × 1 vector representing the fixed effects of genetic variables. We assume that $\overset{\sim }{\varepsilon }$has zero-mean and $Var\left(\overset{\sim }{\varepsilon}\right)={\sigma}_e^2{I}_n$. Note that the participants are independent under linear model (5), while the participants are correlated via the kinship coefficient matrix under mixed model (1).

We first describe the HOLP procedure for the linear model. Under linear model (5), if dimension *p* were small compared with sample size *n*, we could consider the following least-squares (LS) estimate,

6 $$ {\overset{\sim }{\beta}}_{LS}={\left({\overset{\sim }{X}}^{\prime}\overset{\sim }{X}\right)}^{-1}{\overset{\sim }{X}}^{\prime}\overset{\sim }{Y} $$

But for the setting where *p* ≫ *n*, the LS estimate is not applicable owing to the aforementioned curse of dimensionality. To overcome this problem, the HOLP procedure [10] simply rearranges the positions of design matrix $\overset{\sim }{X}$ in equation (6) and uses the following estimate:

7 $$ {\overset{\sim }{\beta}}_{JS}={\overset{\sim }{X}}^{\prime }{\left(\overset{\sim }{X}{\overset{\sim }{X}}^{\prime}\right)}^{-1}\overset{\sim }{Y} $$

Equations (6) and (7) are commonly known as “dual equations”; see, for example, Shawe-Taylor and Cristianini [11]. Equation (7) not only solves the problem that the solution to equation (6) is not unique when the dimensional of variables is high, but also, more importantly, provides some ranking for those variables. That is, based on ${\overset{\sim }{\beta}}_{JS}$, we can conduct JS, using the following subset of variables for the second stage analysis:

8 $$ {\overset{\sim }{\mathcal{M}}}_d=\left\{j:\left|{\overset{\sim }{\beta}}_j\right| is\ among\ the\  top\ d\  ofall\ |{\overset{\sim }{\beta}}_j|\right\} $$

To derive the sure screening consistency of the proposed JS procedure for linear models, Wang and Leng [10] assumed that the true coefficient vector *β* in equation (5) is sparse; that is, many of the components of *β* are exactly equal to zero. Let ${\mathcal{M}}_{\ast }=\left\{j:{\beta}_j\ne 0\right\}$, where *β* is the true coefficient vector in equation (5). Wang and Leng showed that, under some standard conditions on the design matrix $\overset{\sim }{X}$ and some weak condition on *d*,$Prob\ \left({\mathcal{M}}_{\ast}\subseteq {\overset{\sim }{\mathcal{M}}}_d\right)\to 1$ as *n* → ∞ and *p* diverges with *n*. Furthermore, under some condition on *d*,$Prob\ \left({\overset{\sim }{\mathcal{M}}}_d={\mathcal{M}}_{\ast}\right)\to 1$ as *n* → ∞ and *p* diverges with *n*.

Now we are ready to describe our JS procedure for mixed models. Assume for the moment that the covariance matrix *V* given by equation (2) is known. Under the transformation $\overset{\sim }{Y}={V}^{-1/2}Y,$ mixed model (1) becomes

$$ \overset{\sim }{Y}={V}^{-1/2} X\beta +{V}^{-1/2}\left(\ \alpha +\varepsilon\ \right)=\overset{\sim }{X}\beta +\overset{\sim }{\varepsilon } $$

which is equivalent to linear model (5). Therefore, motivated by the idea of HOLP in equation (7), we propose the JS estimate for a mixed model as ${\overset{\sim }{\beta}}_{JS}={\overset{\sim }{X}}^{\prime }{\left(\overset{\sim }{X}{\overset{\sim }{X}}^{\prime}\right)}^{-1}\overset{\sim }{Y}$, where $\overset{\sim }{Y}={V}^{-1/2}Y,$and $\overset{\sim }{X}={V}^{-1/2}X$. Now, if we plug in the transformations into the above equation, we have

$$ {\overset{\sim }{\beta}}_{JS}={X}^{\prime }{V}^{-1/2}{\left({V}^{-1/2}X{X}^{\prime }{V}^{-1/2}\right)}^{-1}{V}^{-1/2}Y $$

$$ ={X}^{\prime }{V}^{-1/2}{V}^{1/2}{\left(X{X}^{\prime}\right)}^{-1}{V}^{1/2}{V}^{-1/2}Y $$

$$ ={X}^{\prime }{\left(X{X}^{\prime}\right)}^{-1}Y $$

Therefore, under mixed model (1), the JS estimate is

9 $$ {\widehat{\beta}}_{JS}={X}^{\prime }{\left(X{X}^{\prime}\right)}^{-1}Y $$

For the rest of the article we denote the JS estimate for the mixed model (1) by ${\widehat{\beta}}_{JS}$to differentiate it from the linear model estimategiven by equation (7). It is important to note that the JS screening estimate (9) does not depend on unknown matrix *V*. Thus, we avoid the computationally difficult problem of estimating *V* via the REML (4). Because the matrix under inverse in equation (9), *XX*^′^, is an *n* × *n* matrix, the computation of equation (9) is computationally fast for the settings where *p* ≫ *n*. The estimate for equation (9) has a computational complexity of $\mathcal{O}\left({n}^2p\right)$.

Based on ${\widehat{\beta}}_{JS}$, we can conduct JS for mixed model (1); that is, consider subset

10 $$ {\hat{\mathcal{M}}}_d=\left\{j:|{\hat{\beta}}_j|\mathrm{is}\ \mathrm{among}\ \mathrm{the}\ \mathrm{top}\ d\ \mathrm{of}\ \mathrm{all}\ |{\hat{\beta}}_j|\right\} $$

and use it for the second stage analysis. We assume that the true coefficient vector *β* is sparse. Let ${\mathcal{M}}_{\ast }=\left\{j:{\beta}_j\ne 0\right\}$, where *β* is the true coefficient vector in equation (1). By similar arguments in Wang and Leng [10], we can derive the sure screening consistency of the proposed JS procedure for mixed models, under those conditions in Wang and Leng [10] plus an extra condition that there exists *τ* ≥ 0 and *c* > 0 such that *λ*_max_(*V*)/*λ*_min_(*V*) ≤ *cn*^*τ*^, where *λ*_max_(*V*) and *λ*_min_(*V*) are the maximum and minimum eigenvalues of *V*. That is, under some standard conditions on the design matrix *X* and some weak condition on *d*,$Prob\ \left({\mathcal{M}}_{\ast}\subseteq {\widehat{\mathcal{M}}}_d\right)\to 1$ as *n* → ∞ and *p* diverges with *n*. Furthermore, under some condition on *d*,$Prob\ \left({\widehat{\mathcal{M}}}_d={\mathcal{M}}_{\ast}\right)\to 1$ as *n* → ∞ and *p* diverges with *n*.

#### Determination of *d*

The determination of *d* is an important issue. Here we describe two common approaches. One approach is that we use a conservatively large *d* initially, say *d* = *n*. Then, based on the top *d* genetic variables, we apply some penalized mixed model, say the *l*_1_-penalized mixed model [9] along with 10-fold cross-validation, to select a participant of *d*^′^ genetic variables, where *d*^′^ < *d*. Another approach is that we simply use *d* = ⌊*n*/ log(*n*)⌋. This approach was first considered by Fan and Lv [3], where they proposed the SIS procedure. In this article, because we propose a two-stage strategy to analyze the GAW20 data, we consider the second approach to determine the value of *d*; that is, *d* = ⌊*n*/ log(*n*)⌋.

#### A simulation study

We conduct a simulation study to demonstrate that the proposed JS procedure for mixed models is robust to the familial effects. Consider the following model:

$$ {\mathrm{y}}_{ij}={\alpha}_i+{\mathrm{x}}_{ij}^{\prime}\beta +{\varepsilon}_{ij},i=1,\cdots, 100;j=1,\cdots, 5 $$

The values of the parameters are taken to be:

$$ \left(p,n\right)=\left(100000,500\right); $$

(i). There are 100 families; each has 5 participants;

$$ {\alpha}_i\sim N\left(0,{\sigma}^2\right),{\mathrm{x}}_{ij}\sim MVN\left(0,{I}_n\right); $$

$$ \beta =\left(5.2,-\mathrm{4.5,0.9,2.1},-\mathrm{3.8,0},\cdots, 0\right); $$

$$ {\sigma}^2=\left\{\mathrm{0,0.1,0.2,0.5,1},2,5\right\}. $$

We examined the properties of ${\widehat{\beta}}_{JS}$ in equation (9) for different values of *σ*^2^. For the JS screening estimate of equation (9) to be robust, the percent of times the nonzero *β* appears in the largest *d* ( = ⌊*n*/ log(*n*)⌋ = ⌊500/ log(500)⌋ = 80) ${\widehat{\beta}}_{JS}$ should not vary much. In fact, the percent of nonzero *β* hovers around 84% for the chosen *σ*^2^.

This shows us that the proposed estimator (9) is insensitive toward the covariance structure of the random effects. Having discovered this important property of the HOLP estimator, we proceed to apply it to the GAW20 data set.

### Stage 2: Analysis on the selected ***d*** variables

The JS stage selects *d* genetic variables. An advantage of our JS procedure over the existing marginal screening is that the selected *d* genetic variables are expected to be highly associated with the outcome variable. Now in the second stage, we can apply mixed models to analyze the associations between these selected genetic variables and the outcome variable. Because we have reduced the number of variables to be within a manageable range, say *d* < *n*, it is straightforward to implement mixed model analysis using existing statistical software such as R and SAS.

Specifically, we consider mixed model (1), where there is one individual random effect for each participant; that is, *α*_*i*_ for participant *i*. The correlations among *α*_*i*_ are quantified using the kinship coefficient matrix *K*. The kinship coefficient matrix can be computed easily by knowing the father ID and mother ID for each participant. Actually, participants are only correlated within each pedigree, and participants from different pedigrees are uncorrelated. Therefore, *K* is a diagonal blockmatrix, and the implementation of mixed model analysis is computationally fast.

In this stage, we can conduct statistical inferences using the results from the mixed model analysis. We can examine the effect size of each genetic variable. We can also test the statistical significance for each genetic variable. Because there are *d* genetic variables under the consideration, we should consider multiple-comparison correction when we explain the statistical testing results. For example, we can consider the false discovery rate control. We can also consider the Bonferroni correction, using *α* = 0.05/*d* as the significance level to claim significance findings.

The numerical results were obtained using software SAS 9.4. We used SAS procedure PROC IML for Matrix calculations and PROC INBREED to compute the kinship matrix *K*. We conducted mixed model analysis using PROC MIXED.

## Results

### Computational cost

The sample size is *n* = 680, as only 680 out of 717 subjects participants have TG-level data at visit 4. At the screening stage, to screen *p* = 463,995 CpG sites, the computation of the JS estimate, ${\widehat{\beta}}_{JS}$, took approximately 12 minutes on an Intel® Core™ i7-7500 U 2.70GHz, 2901 Mhz Processor. At the second stage, the computation time to apply PROC MIXED on *d* = ⌊680/ log(680)⌋ = 104 variables is ignorable.

### Results from stage 1

We perform the proposed JS procedure to identify significant CpG sites. We consider the difference in the TG level between visit 4 and visit 2 as the outcome variable. Accordingly, we also consider the differences in the CpG sites between visit 4 and visit 2 as the predictors, as both the TG level and the CpG value change as time goes by. That is, we consider

11 $$ {\displaystyle \begin{array}{ll}Y& = TG{L}_4- TG{L}_2,\\ {}{X}_j& = Cp{G}_4- Cp{G}_2,j=1,\cdots, p.\end{array}} $$

**Fig. 2:**
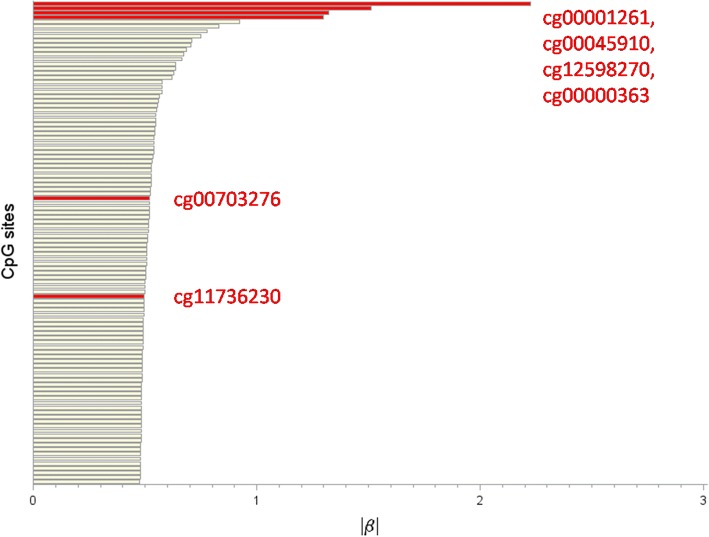
Screening results. The plot shows the ${\widehat{\beta}}_{JS}$ estimates from the JS procedure under equation (11)

We compute the JS estimate (9), using the GAW20 representative simulated data set with *n* = 680 observations and *p* = 463,995 CpG sites. We specify *d* = ⌊680/ log(680)⌋ = 104 and we obtain the select subset (8). We observe from Fig. 2 that among the truly significant CpGs used in generating the simulated data, *cg00001261*, *cg00045910*, *cg12598270*, *cg00000363*, *cg00703276*, and *cg11736230* passed the screening.

### Results from stage 2

We perform mixed model analysis (1), using the GAW20 representative simulated data set with *n* = 680 observations and *d* = 104 selected genetic variables plus other important risk factors, namely, age, gender, smoking, and metabolic syndrome.

First we conduct residual diagnostics using conditional Pearson residuals to check the goodness of fit of the above mixed model using CpG sites as variables. We observed that the residuals approximately follow normal distribution, which indicates the model is appropriate. Residual plots have been omitted because of space restrictions.

Table 1 shows the mixed model results from the second stage. However, as can be observed from the table, none of the CpG sites used for simulating the data became significant at the 5% level.

**Table 1:** Solutions for fixed effects for CpG sites

Effect	Chr# (BP)	Estimate	*p*-Value
Intercept	–	−26.27	< 0.0001
ATP meta syn^a^	–	− 34.32	< 0.0001
*cg01606628*	6(3063768)	− 175.41	0.0003
*cg01929239*	2(114346218)	− 184.82	0.0002
*cg01965874*	1(19052204)	−65.82	0.0175
*cg02317738*	5(7847407)	− 137.66	0.0093
*cg02586268*	1(173883567)	− 242.13	<.0001
*cg02985292*	16(687604)	191.16	<.0001
*cg04404270*	1(151508741)	− 106.60	0.0007
*cg05653055*	17(20841843)	−90.22	0.0005
*cg06653026*	7(84892267)	76.05	0.0162
*cg07741992*	8(95303464)	137.41	0.0031
*cg07748719*	16(1272498)	− 119.45	0.0109
*cg08711796*	22(16287910)	185.82	0.0001
*cg11016563*	11(101454626)	148.60	0.0016
*cg11725972*	7(155191845)	−162.89	0.0003
*cg14518098*	9(135085065)	152.69	<.0001
*cg14553506*	3(183957794)	25.57	0.0116
*cg14710552*	7(134832584)	99.04	0.0109
*cg15155441*	11(57005981)	149.42	0.0003
*cg15399174*	15(28349794)	− 187.49	0.0005
*cg15469014*	19(11032172)	204.24	0.0037
*cg16776885*	12(132834399)	44.93	0.0059
*cg16893574*	16(71392095)	153.00	0.0015
*cg17661462*	19(7741838)	−177.99	0.0002
*cg18320647*	14(61201977)	101.74	0.0056
*cg18473686*	22(33427086)	119.85	0.0072
*cg19057882*	20(37101373)	99.74	0.047
*cg19191624*	2(32582276)	−154.06	0.0014
*cg19425116*	19(57804150)	−63.71	0.0327
*cg20929733*	1(1572082)	−105.13	0.0031
*cg20933109*	14(36991034)	126.93	0.0102
*cg21397592*	8(23167206)	176.20	0.0009
*cg22171993*	5(81818816)	133.09	0.0045
*cg22610434*	1(158259914)	−40.34	0.0371
*cg22848704*	1(48648439)	200.79	0.0001
*cg23774356*	22(19137874)	−105.17	0.0114
*cg23968558*	17(17583879)	−162.86	0.0007
*cg24332389*	1(6558085)	160.98	< 0.0001
*cg24805360*	5(77930038)	85.76	0.0021
*cg24973221*	10(134407873)	−83.08	< 0.0001
*cg25371129*	6(31599646)	131.33	0.0031
*cg25826973*	6(31865892)	− 111.47	0.0085
*cg26685197*	1(10003173)	− 198.32	0.0004
*cg27087233*	6(138860932)	−146.65	0.0003

^a^metabolic syndrome defined by ATP

## Discussion

Mixed models are a useful tool for analyzing family data. But when the dimension of the genetic variables is ultrahigh, it is computationally difficult to fit mixed models, and the results from any fitted mixed model will be unstable. To overcome this problem, we can consider a two-stage strategy; in the first stage we perform variable screening and in the second stage we conduct regular mixed model analysis on a manageable number of variables that pass the screening.

In this article, we propose a novel JS procedure for the first stage. It is novel because the existing screening procedures are marginal, like the one used by Irvin et al. [2].

While marginal screening procedures fit a mixed model for one genetic variable at a time, the proposed JS procedure considers all the genetic variables simultaneously. As high-dimensional data tend to have correlated predictors, marginal screening procedures may select unimportant variables that have a high degree of association to important predictors. Likewise, these procedures may fail to select truly important variables that are jointly correlated but have no marginal association to the response. The proposed JS procedure is efficient at detecting both marginally and jointly significant variables.

We performed screening using the outcome variables as defined by equation (11) and selected a subset of 104 genetic variables. As the TG-level values are skewed, it is advisable to do a log-transformation so that normality assumption is not violated. In contrast, the JS screening procedure performs well under nonnormality of the outcome variable. Also, it makes sense to consider the difference in CpG values, if we are using them for the outcome variable. We have shown that screening using equation (11) performs well, as 6 out of the 10 truly significant variables pass the screening.

## Conclusions

We consider a two-stage strategy for fitting mixed models to family data with ultrahigh dimensional variables. We propose a novel JS procedure to identify a manageable subset of variables. The proposed procedure is computationally efficient and enjoys the desirable sure screening consistency. Application to the GAW20 data shows that the proposed JS procedure performs well.

However, the proposed two-stage strategy considers screening and testing on the same data, and the users should be cautioned that it may inflate the family-wise error [12]. If the data set is large, we could divide the data into two halves, one for screening and one for testing. The impact of this two-stage strategy on the family-wise error is not investigated here and would be investigated in future work.
